# The long non-coding RNA NONHSAG026900 predicts prognosis as a favorable biomarker in patients with diffuse large B-cell lymphoma

**DOI:** 10.18632/oncotarget.16163

**Published:** 2017-03-13

**Authors:** Shuangtao Zhao, Shuangsang Fang, Yanhua Liu, Xixi Li, Shengyou Liao, Jinwen Chen, Jingjia Liu, Lianhe Zhao, Hui Li, Wei Zhou, Wenzhi Shen, Xiaoli Dong, Rong Xiang, Luhua Wang, Yi Zhao

**Affiliations:** ^1^ Department of Radiation Oncology, National Cancer Center/Cancer Hospital, Peking Union Medical College and Chinese Academy of Medical Sciences, Beijing, China; ^2^ The Key Laboratory of Intelligent Information Processing, Institute of Computing Technology, Chinese Academy of Sciences, Beijing, China; ^3^ The School of Medicine, Nankai University, Tianjin, China; ^4^ The Collaborative Innovation Center for Biotherapy, Nankai University, Tianjin, China; ^5^ The Tianjin Key Laboratory of Tumor Microenvironment and Neurovascular Regulation, Tianjin, China; ^6^ Department of Pathology, Nankai University, Tianjin, China

**Keywords:** diffuse large B-cell lymphoma, long non-coding RNA, NONHSAG026900, prognosis, biomarker

## Abstract

Long non-coding RNAs are known to be involved in cancer progression, but their biological functions and prognostic values are still largely unexplored in diffuse large B-cell lymphoma. In this study, long non-coding RNAs expression was characterized in 1,403 samples including normal and diffuse large B-cell lymphoma by repurposing 7 microarray datasets. Compared with any stage of normal B cells, NONHSAG026900 expression was significantly decreased in tumor samples. And in germinal center B-cell subtype, the significantly higher expression of NONHSAG026900 indicated it was a favorable prognosis biomarker. Then the prognostic power of NONHSAG026900 was validated with another independent dataset and NONHSAG026900 improved the predictive power of International Prognostic Index as an independent factor. Moreover, functional prediction and validation demonstrated that NONHSAG026900 could inhibit cell cycle activity to restrain tumor proliferation. These findings identified NONHSAG026900 as a novel prognostic biomarker and offered a new therapeutic target for diffuse large B-cell lymphoma patients.

## INTRODUCTION

Diffuse large B-cell lymphoma (DLBCL) is biologically heterogeneous and accounts for 30–35% [1] of all non-Hodgkin lymphomas (NHLs). The standard chemotherapy regimen of CHOP (cyclophosphamide, doxorubicin, vincristine, and prednisone), especially R-CHOP (rituximab, cyclophosphamide, doxorubicin, vincristine, and prednisone), has significantly improved the survival of patients with DLBCL [2, 3]. However, approximately one-third of patients will relapse shortly after initial remission and eventually succumb to this refractory disease [4–6]. In clinical practice, the International Prognostic Index (IPI) does not fully represent DLBCL heterogeneity, despite its status as one of the most important clinical prognosis predictors [7]. Therefore, some novel prognostic factors are being explored to help predict treatment outcome.

Long non-coding RNAs (lncRNAs) are defined as non-protein-coding RNAs of more than 200 nucleotides in length [8]. Although lacking protein coding capability, lncRNAs were reported as biomarkers for predicting prognosis, metastasis, and in multiple disease diagnosis [9–12]. It is possible to detect the quantity of lncRNAs because of the relative stability of their secondary structures in the body [13]. Many lncRNAs are reported to be important in regulating cancer cell proliferation, invasion, and metastasis [14, 15]. Peng *et al*. reported that lincRNA-p21 could predict favorable clinical outcome and impair tumorigenesis in DLBCL patients with an R-CHOP regimen [16]. However, the biological functions and prognostic value of lncRNAs in DLBCL are still largely unexplored.

In our study, we investigated lncRNA functions in 7 GEO databases, including five differentiated stages of normal B cells (naive B cells, centroblasts, centrocytes, memory B cells, and plasma cells) and DLBCL samples. Our research identified that NONHSAG026900 was significantly down-regulated in DLBCL samples, and could serve as a favorable biomarker to predict prognosis of DLBCL patients. Additionally, we integrated protein-coding gene expression into a co-expression network to predict the possible function of NONHSAG026900.

## RESULTS

### Transcription analysis and the differential regulation of lncRNAs between normal and DLBCL samples

**Figure 1:**
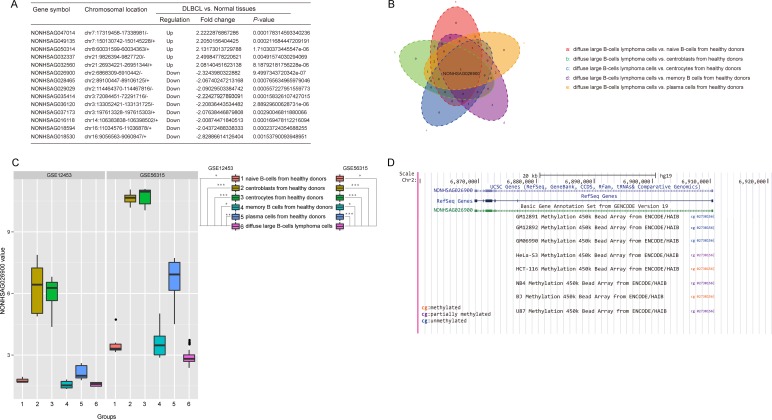
Screening the significant lncRNAs that were differentially expressed between normal and DLBCL tissues (**A**) A total of 5 lncRNAs were up-regulated, while 9 lncRNAs were down-regulated, in DLBCL compared with normal tissues (Fold change > 2.0, *P* < 0.05, *t*-test method). (**B**) A Venn diagram revealed the overlapping genes among the five groups which were composed of the significantly different genes between DLBCL cells and normal B cells in each differentiation step. *P* values were calculated with a one-way ANOVA method (Bonferroni correction). (**C**) Boxplots revealed that the expression of NONHSAG02900 was significantly down-regulated in DLBCL compared with normal tissues in GSE12453 and GSE56315 (*P* < 0.05, one-way ANOVA method). (**D**) Genomic context of NONHSAG026900. A CpG methylation site was discovered upstream of the NONHSAG026900 coding sequence.

Utilizing the published GEO dataset GSE12453 we concentrated on repurposing the data, including 11 DLBCL samples and 25 normal B cells samples represented five differentiated stages (naive B cells, centroblasts, centrocytes, memory B cells and plasma cells). We applied ncFANs software to re-annotate all the collected probes in the Affymetrix microarray platforms (HGU 133 plus 2.0). The probes were stratified into two groups comprising 14,707 protein-coding genes and 6,307 lncRNAs in GSE12453. We then reanalyzed the expression profiles between normal samples and DLBCL samples. As a result, we obtained 226 protein-coding genes (Supplementary Table 1) and 14 lncRNA genes (Figure 1A) with significantly different expressions (*P* < 0.05, and Fold change > 2).

Because DLBCL might occur at any stage of normal B cell development, we compared the 14 lncRNAs expression among the five stages of normal B cell differentiation and the DLBCL stage with one-way ANOVA (with Bonferroni correction). Thus, we found that NONHSAG026900 was the common significantly alterative lncRNA (*P* = 0.000, Figure 1B) and it was down-regulated significantly in DLBCL samples (*P* = 0.000, Figure 1C).

To confirm our findings, we validated the expression profile of NONHSAG026900 in another data set GSE56315, which consisted of 33 normal samples with five different stages and 74 DLBCL samples. Consistent with the result above, NONHSAG026900 was significantly lower expression in DLBCL samples than the other normal groups (*P* = 0.000, Figure 1C). These results demonstrated that a decreased expression of NONHSAG026900 might be associated with the presence of DLBCL.

### Potential NONHSAG026900 transcriptional control mechanism

To explore the mechanism of much lower expression of NONHSAG026900 in DLBCL than normal, we identified a CpG methylation site 146 bp upstream of the transcription start site for NONHSAG026900 in 8 cell lines (GM12891, GM12892, GM06990, HeLa-S3, HCT-116, NB4, BJ, and U87; Figure 1D) by using the ENCODE database. The data revealed that there were no methylated sites in normal B lymphocyte cell lines (GM12891, GM12892, and GM06990), whereas partially or completely methylated sites were found in human B-cell lymphoma cell lines (BJ) and four other cancer cell lines: a cervical cancer cell line (HeLa-S3), a colon cancer cell line (HCT-116), a leukemia cell line (NB4), and a brain star glioblastoma cell line (U87). Because a promoter with DNA methylation could suppress the expression of the gene under the control of that promoter [17], this result could partially explain the low levels of NONHSAG026900 expression in tumor tissues. However, these discoveries should be validated by experimentation.

### Identification of diagnostic power from the NONHSAG026900 value distribution

**Figure 2:**
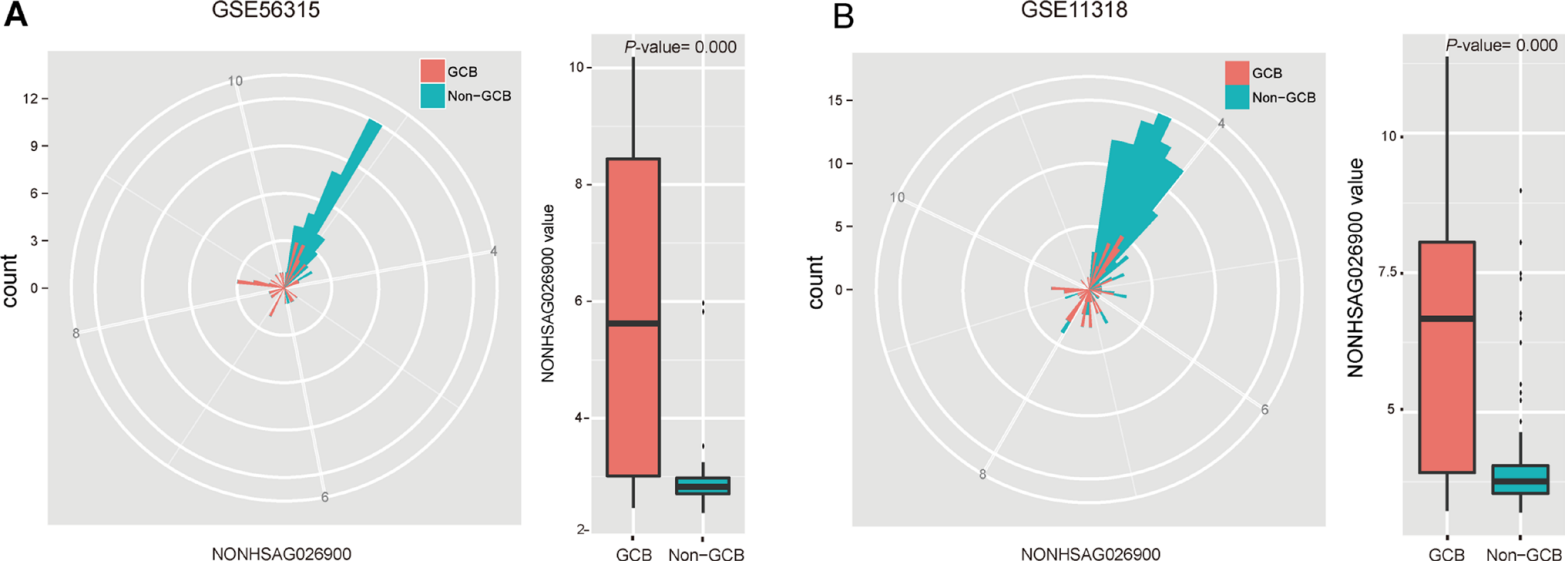
Analysis of the predictive power of NONHSAG026900 from the distribution of its expression values (**A**) The radar map method and boxplot showed that the value of NONHSAG026900 was significantly greater in the GCB than non-GCB subgroup in GSE56315 (*P* = 0.000, *t*-test method). (**B**) The radar map method was used for validating that the distribution of NONHSAG026900 expression values between GCB and non-GCB subgroups in GSE11318 was similar to that in GSE56315, while the boxplot analysis demonstrated that the value of NONHSAG026900 was significantly lower in the non-GCB subtype DLCBL patients in GSE11318 (*P* = 0.000, *t*-test method).

To further explore the expression patterns of NONHSAG026900, we performed a deeper analysis of its distribution between GCB and non-GCB subtypes in 51 patients with DLBCL from GSE56315. Our analysis indicated that patients with GCB subtype had significantly higher values of NONHSAG026900 than non-GCB subtype (*P* = 0.000, Figure 2A). Similarly, we confirmed the result above in GSE11318 (*n* = 170, *P* = 0.000, Figure 2B). So, we concluded NONHSAG026900 might have diagnostic potential deduced by its distribution status between GCB and non-GCB subgroups. Also, because the GCB-DLBCL patients were reported to have a more favorable outcome than those with non-GCB subtype [18], we inferred that NONHSAG026900 could act as a favorable biomarker of prognosis in DLBCL patients.

### Identification of prognostic power from the NONHSAG026900 value distribution

**Figure 3:**
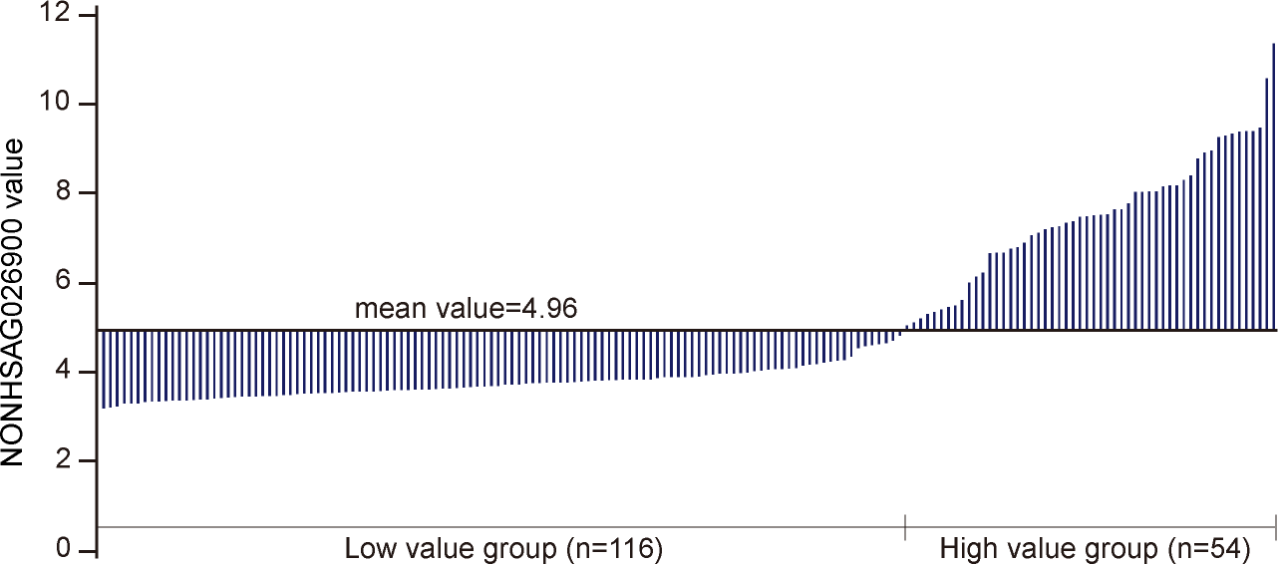
Divide the NONHSAG026900 lncRNA levels into two groups Stratification of the 170 DLBCL patients from GSE11318 into two groups (low value group, *n* = 116 and high value group *n* = 54) via the mean value of NONHSAG026900 expression.

To gain further insights into the prognostic role of NONHSAG026900 in DLBCL we analyzed the association between the NONHSAG026900 expression and clinical features in 170 patients treated with the CHOP regimen from GSE11318. According to the mean value of NONHSAG026900 expression in patients, we classified them into two groups: a low value group (< 4.96, *n* = 116) and a high value group (≥ 4.96, *n* = 54; Figure 3 and Table 1). We discovered that NONHSAG026900 was not correlated with gender (*P* = 0.867), age (*P* = 0.055), Ann Arbor stages (*P* = 0.980), ECOG performance status (*P* = 0.889), LDH ratio (*P* = 0.124), extra nodal sites (*P* = 0.996), or IPI score (*P* = 0.350). However, NONHSAG026900 expression was closely correlated with GCB vs. non-GCB genotype (*P* = 0.000, Table 1).

**Table 1:** Correlation between NONHSAG026900 and clinicopathological characteristics in DLBCL

Characteristics	*n*	NONHSAG026900 expression		*P* -value
		Low value	High value	
**Gender**				0.867
Male	96	65	31	
Female	74	51	23	
**Age (years)**				0.055
< 60	64	38	26	
≥ 60	99	73	26	
**Ann Arbor stages**				0.980
I–II	75	51	24	
III–IV	87	59	28	
**ECOG performance status**				0.889
0-1	122	83	39	
2-4	39	27	12	
**LDH ratio**				0.124
≤ 1	68	40	28	
> 1	76	54	22	
**Extra nodal sites**				0.996
< 2	134	91	43	
≥ 2	28	19	9	
**IPI score**				0.350
0–2	104	66	38	
3–5	39	28	11	
**Genotype**				0.000*
GCB	70	26	44	
Non-GCB	100	90	10	

* Statistically significant (*P* < 0.05), Pearson Chi-Square test.

**Figure 4:**
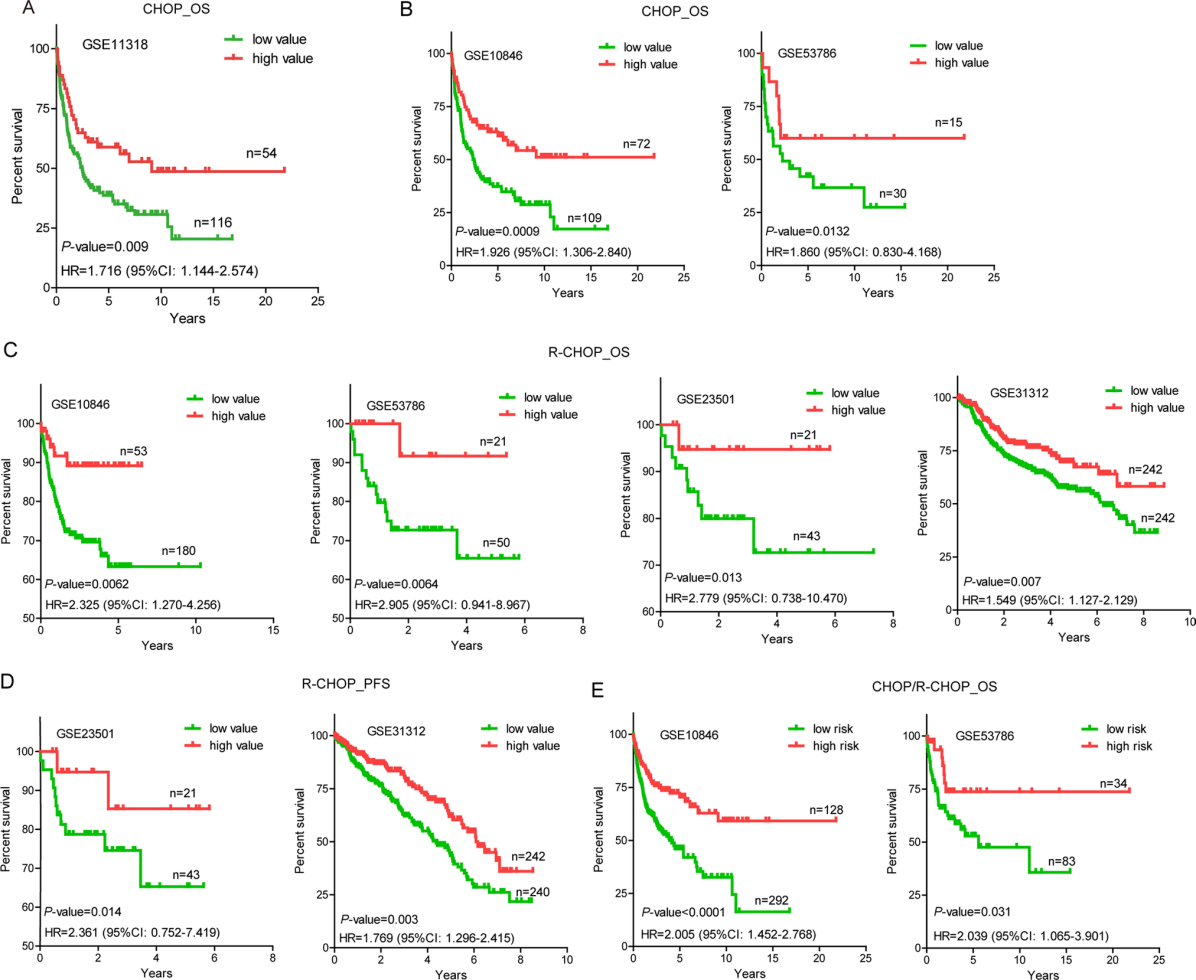
Overall survival (OS) or progression-free survival (PFS) analysis of DLBCL patients with CHOP/R-CHOP regimens in the training and validated cohorts (**A**) A Kaplan-Meier survival curve analysis showed that patients with higher expression of NONHSAG026900 showed increased overall survival (OS) compared with lower expression of NONHSAG026900 in GSE11318. The *P*-value was calculated by a log-rank test. The Kaplan-Meier plots were used to visualized the OS or PFS probabilities for the high value versus low value group of patients based on the threshold value of mean expression. (**B**) OS analysis by Kaplan-Meier curves for GSE10846 (*n* = 181) and GSE53486 (*n* = 45) patients with CHOP regimen; (**C**) OS analysis by Kaplan-Meier curves for GSE10846 (*n* = 233), GSE53786 (*n* = 71), GSE23501 (*n* = 64) and GSE31312 (*n* = 484) patients with R-CHOP regimen; (**D**) PFS analysis by Kaplan-Meier curves for GSE23501 (*n* = 64) and GSE31312 (*n* = 482) patients with R-CHOP regimen; (**E**) OS analysis by Kaplan-Meier curves for all GSE10846 (*n* = 420) and GSE53486 (*n* = 117) patients with CHOP/R-CHOP regimen. The tick marks on the Kaplan-Meier curves represented the censored subjects. The *P*-value was calculated by the two-sided log-rank test.

Next, we performed log-rank tests between the low and high value groups in GSE11318. We found that patients with low value expressions of NONHSAG026900 had higher risk than those with high values (hazard ratio (HR) = 1.716, 95% CI: 1.144–2.574), and that 5-year overall survival (OS) rates (38.67%) in the group with low-NONHSAG026900 were significantly poorer than those (58.82%) in the high-NONHSAG026900 group (*P* = 0.009, Figure 4A). Generally, these results suggested that NONHSAG026900 could be used as a favorable biomarker associated with prognosis in DLBCL patients.

### Validation of NONHSAG026900 for survival prediction in GEO data sets

To confirm our discoveries, we selected another four GEO datasets to validate the prognostic power of NONHSAG026900. Similarly, we stratified patients of each independent cohort into two groups (low and high value) by using the mean values as the cutoff point. In accordance with the results above from patients treated with a CHOP regimen in GSE11318, patients with a CHOP regimen from GSE10846 in the low value group (*n* = 109) had a higher risk (HR = 1.926, 95% CI: 1.306–2.840) than those in the high value group (*n* = 72), while 5-year OS rates (37.28%) in the low value group were significantly poorer than those (61.16%) in the high value group (*P* = 0.0009, Figure 4B). Mean value-based classification of another cohort with a CHOP regimen from GSE53786 (*n* = 45) also produced similar results (Figure 4B). The HR (low vs. high group) in this cohort was 1.860 (95% CI: 0.830–4.168). In addition, 5-year OS rates in the low value group (41.92% vs. 60.00%) were significantly worse compared with the high value group in GSE53786 (*P* = 0.0132, Figure 4B). All these results suggested that NONHSAG026900 could predict survival of DLBCL patients treated with a CHOP regimen, and function as a favorable biomarker.

To validate the prognostic power of NONHSAG026900 in patients with an R-CHOP regimen, four cohorts (GSE10846, *n* = 233; GSE53786, *n* = 71; GSE23501, *n* = 64; and GSE31312, *n* = 484) were included in our study. In these independent subsets of patients who were treated with an R-CHOP regimen we confirmed the reliability of NONHSAG026900 in predicting survival. The mean value classification stratified each group of patients into two subgroups (low and high value groups) with significantly different HRs (HR > 1.5, *P* < 0.05, Figure 4C). Also, patients with low expression values had significantly shorter 5-year OS rates (63.29% vs. 89.15% in GSE10846, *P* = 0.0062; 65.45% vs. 91.67% in GSE53786, *P*=0.0064; 72.68% vs. 94.74% in GSE23501, *P* = 0.013; and 56.97% vs. 69.68% in GSE31312, *P* = 0.007; Figure 4C) or progression-free survival (PFS) rates (65.24% vs. 85.26% in GSE23501, *P* = 0.014; and 43.48% vs. 61.89% in GSE31312, *P* = 0.003; Figure 4D) than those with high value expression in the four validated cohorts.

Next, we combined the two groups of patients with CHOP or R-CHOP regimens into one cohort in GSE10846 (*n* = 420) or GSE53786 (*n* = 117) to verify the predictive power of NONHSAG026900 in clinical outcomes. We obtained a similar result with patients with single CHOP or R-CHOP regimens. Patients in the low value group were at higher risk than those in the high value group (HR = 2.005 (95% CI:1.452–2.768) in GSE10846; and HR = 2.039 (95% CI:1.065–3.901) in GSE53786). 5-year OS rates (46.00% in GSE10846 and 52.91% in GSE53786) in the low value group were significantly worse than those in the high value group (70.46% and 73.77% in GSE10846 and GSE53786, respectively; Figure 4E). Generally, NONHSAG026900 could predict the outcome of DLBCL as a favorable biomarker, no matter what kind of treatments (CHOP or R-CHOP) were selected for patients in clinical practice.

Table 2 revealed that the NONHSAG026900 expression value was significantly associated with OS as a continuous variable in GSE11318 and the four validation cohorts (*P* < 0.05) using a univariate Cox regression analysis. The association of the NONHSAG026900 expression value and OS was also significant (*P* < 0.05, Table 2) when it was measured as a continuous variable in a multivariate analysis with Cox regression including another three prognostic factors including gender, genotype and IPI score (composed of age, Ann Arbor stage, ECOG performance status, LDH ratio, and extra nodal sites). These results confirmed the ability of the NONHSAG026900 in predicting survival as an independent factor.

**Table 2:** Univariate and multivariate analyses of prognostic variables in patients with DLBCL

Variables	Univariate analysis			Multivariate analysis		
	HR	95% CI	*P*–value	HR	95% CI	*P*–value
***GSE11318***						
NONHSAG026900 (low vs. high)	1.716	1.144–2.574	0.009*	0.928	0.809–1.064	0.028*
Gender (Female vs. Male)	0.997	0.673–1.478	0.920			
Genotype (GCB vs. Non–GCB)	0.475	0.321–0.704	0.002*	1.073	0.778–1.478	0.038*
IPI scores (0–2 vs. 3–5)	0.224	0.128–0.391	< 0.0001*	1.718	1.401–2.107	0.000*
***GSE23501***						
NONHSAG026900 (low vs. high)	2.779	0.738–10.470	0.013*	0.660	0.186–2.343	0.025*
Gender (Female vs. Male)	1.211	0.356–4.126	0.759			
Genotype (GCB vs. Non–GCB)	0.288	0.095–0.876	0.028*	2.944	0.828–10.469	0.051
IPI scores (0–2 vs. 3–5)	1.498	0.466–0.482	0.497			
***GSE53786***						
NONHSAG026900 (low vs. high)	2.039	1.065–3.901	0.031*	0.967	0.714–1.311	0.031*
Gender (Female vs. Male)	0.994	0.543–1.817	0.788			
Genotype (GCB vs. Non–GCB)	0.463	0.247–0.868	0.016*	1.752	0.589–5.210	0.122
IPI scores (0–2 vs. 3–5)	0.274	0.149–0.504	< 0.0001*	2.276	1.627–3.184	0.000*
***GSE10846***						
NONHSAG026900 (low vs. high)	2.005	1.452–2.768	< 0.0001*	1.021	0.625–1.670	0.010*
Gender (Female vs. Male)	0.918	0.608–1.386	0.954			
Genotype (GCB vs. Non–GCB)	0.497	0.333–0.741	0.000*	2.123	1.341–3.363	0.001*
IPI scores (0–2 vs. 3–5)	0.270	0.178–0.411	0.000*	2.679	1.880–3.816	0.000*
***GSE31312***						
NONHSAG026900 (low vs. high)	1.549	1.127–2.129	0.007*	1.549	1.127–2.129	0.007*
Gender (Female vs. Male)	1.010	0.745–1.371	0.948			
Genotype (GCB vs. Non–GCB)	0.915	0.675–1.241	0.567			
IPI scores (0–2 vs. 3–5)	1.324	0.980–1.791	0.068			

Abbreviations: HR: hazard ratio; CI: confidence interval *Statistically significant (*P* < 0.05 ).

### NONHSAG026900 is superior to other biomarkers and adds to the predictive power of the IPI

**Figure 5:**
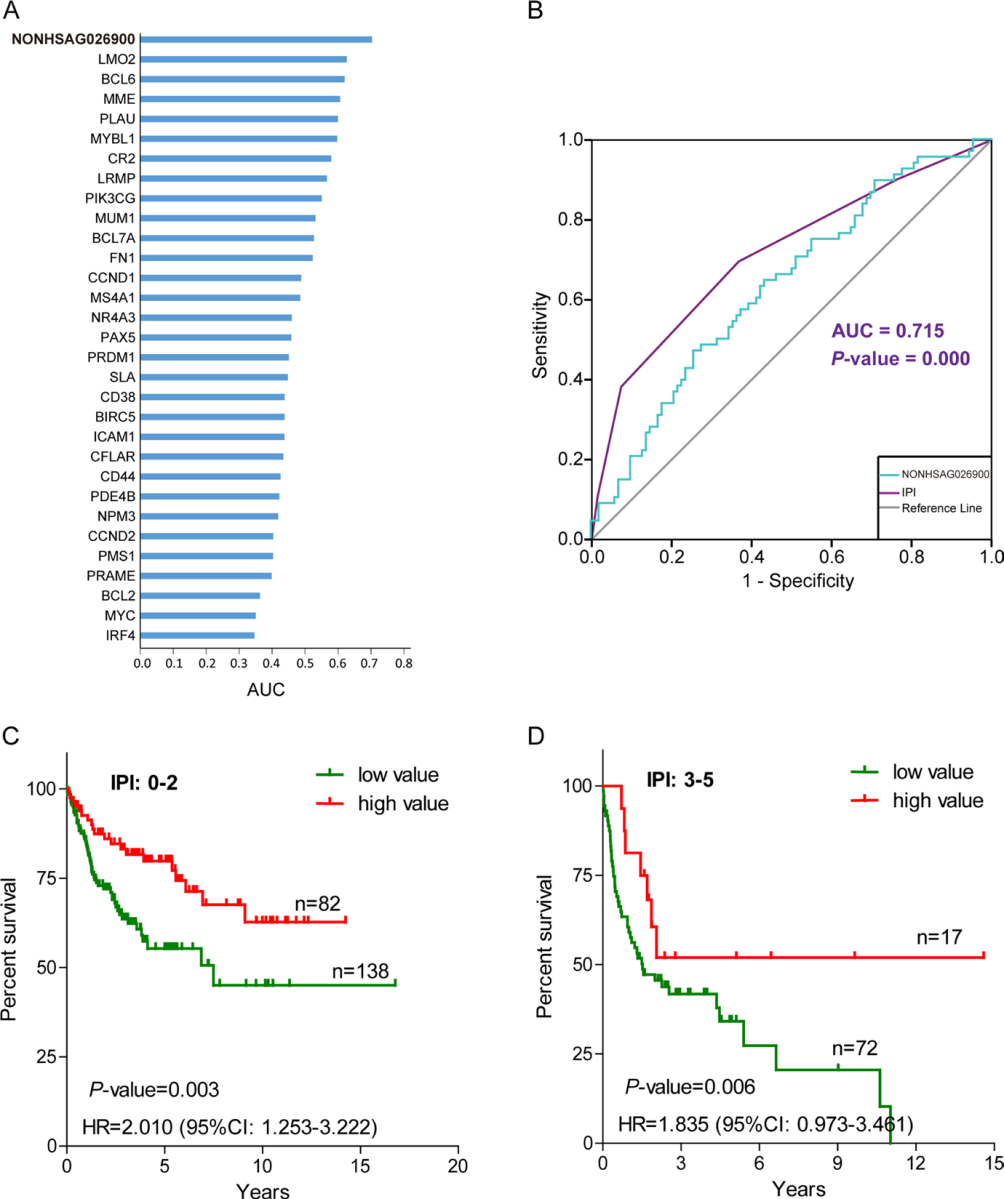
The prognostic power of NONHSAG026900 compared with other predictive biomarkers and the International Prognostic Index (IPI) (**A**) A bar plot was used to show that the area under curve (AUC) value of NONHSAG026900 was greater than 30 other prognostic biomarkers reported previously; (**B**) Receiver operating characteristic (ROC) curve analysis was employed to compare the predictive power between NONHSAG026900 (AUC = 0.703, *P* = 0.003) and IPI (AUC = 0.715, *P* = 0.000); (**C** and **D**) The Kaplan-Meier curves showed overall survival for groups of patients with low IPI scores (0–2) and high IPI scores (3–5) after classification into the low value group or high value group on the basis of cutoff values with the mean. According to log-likelihood estimates, *P* = 0.003 (C) and *P* = 0.006 (D) for the model based on a continuous variable applied to the low and high value groups shown in the figure, respectively.

Multivariate Cox regression showed that NONHSAG026900 expression levels acted as an independent prognostic factor in DLBCL patients. A ROC curve was produced to compare the prognostic power of NONHSAG026900 expression to 30 prognostic biomarkers reported publicly [19–24] in 170 DLBCL patients from GSE11318. The area under the ROC curve (AUC) was 0.703 (*P* = 0.003, Figure 5A), which indicated that the NONHSAG026900 had more diagnostic power for DLBCL than these other 30 biomarkers. Unfortunately, the predictive power of NONHSAG026900 was slightly inferior to the IPI (AUC = 0.715, *P* = 0.000; Figure 5B).

Next, we investigated whether NONHSAG026900 could add to the predictive power of the IPI. We divided patients into two groups (low IPI: 0–2 and high IPI: 3–5) based on the IPI scores. Because there were too few (only 39) patients in the high IPI group from GSE11318 to achieve statistical significance, we selected GSE10846 as an analysis dataset to perform stratification. When DLBCL patients with low IPI scores were divided into two groups per NONHSAG026900 expression values, the HR (low vs. high value group) was 2.010 (95% CI: 1.253–3.222; Figure 5C). Also, 5-year OS rates (55.27%, *n* = 138) in the low value group had significantly worse prognosis than those (79.83%, *n* = 82) in the high value group (*P* = 0.003; Figure 5C). We obtained similar results from the high IPI score group, with a significant HR value (1.835, low vs. high value group; 95% CI: 0.973–3.461) and 5-year OS rates (34.16% vs. 51.95%; *P* = 0.006; Figure 5D). Overall, we could identify 67.96% of all subjects as especially short survival DLBCL patients by evaluating individuals who had low or high IPI scores along with a low-value expression profile (the green line) of NONHSAG026900. Taken together, all of these results suggested that NONHSAG026900 could add to the prognostic power of the IPI as an independent factor.

### Prediction of NONHSAG026900 function

We had identified NONHSAG026900 expression patterns as having diagnostic and prognostic value in DLBCL and therefore wanted to know its biological function. To identify the function of NONHSAG026900 and its potential role in DLBCL pathogenesis we utilized the gene expression profiles from 11 DLBCL samples to develop a coding-non-coding co-expression network. The co-expression network was composed of 856 lncRNA genes and 3,998 coding genes with 9,518 noncoding-noncoding edges, 113,085 coding-coding edges and 25,641 noncoding-coding edges. We predicted NONHSAG026900 functions via two different methods (a module-based analysis and a hub-based analysis). Primarily, a total of 48 module-based subnetworks were produced from the co-expression network by using an MCL algorithm. There were 18 of these modules with at least one enriched GO term. Through parsing the co-expression network into different hub-based subnetworks, we observed 457 lncRNA centered subnetworks with GO term enrichment. Our results demonstrated that NONHSAG026900 was classified into the same module with 134 noncoding genes and 1,174 coding genes.

Because we could infer that genes in the same co-expressed module had similar functions, the role of NONHSAG026900 might be closely associated with those 1,174 coding genes. Therefore, we concluded that the functions of NONHSAG026900 were probably involved with mitosis and cell cycle progression (Supplementary Figure 1, and Supplementary Table 2). Subsequently, we re-analyzed the potential functions of NONHSAG026900 with hub-based analysis, and we discovered that it was surrounded by 55 protein-coding genes (Supplementary Figure 2). In line with the predictions of the module-based method, NONHSAG026900 was predicted to suppress mitotic cell cycle (Supplementary Table 3).

**Figure 6:**
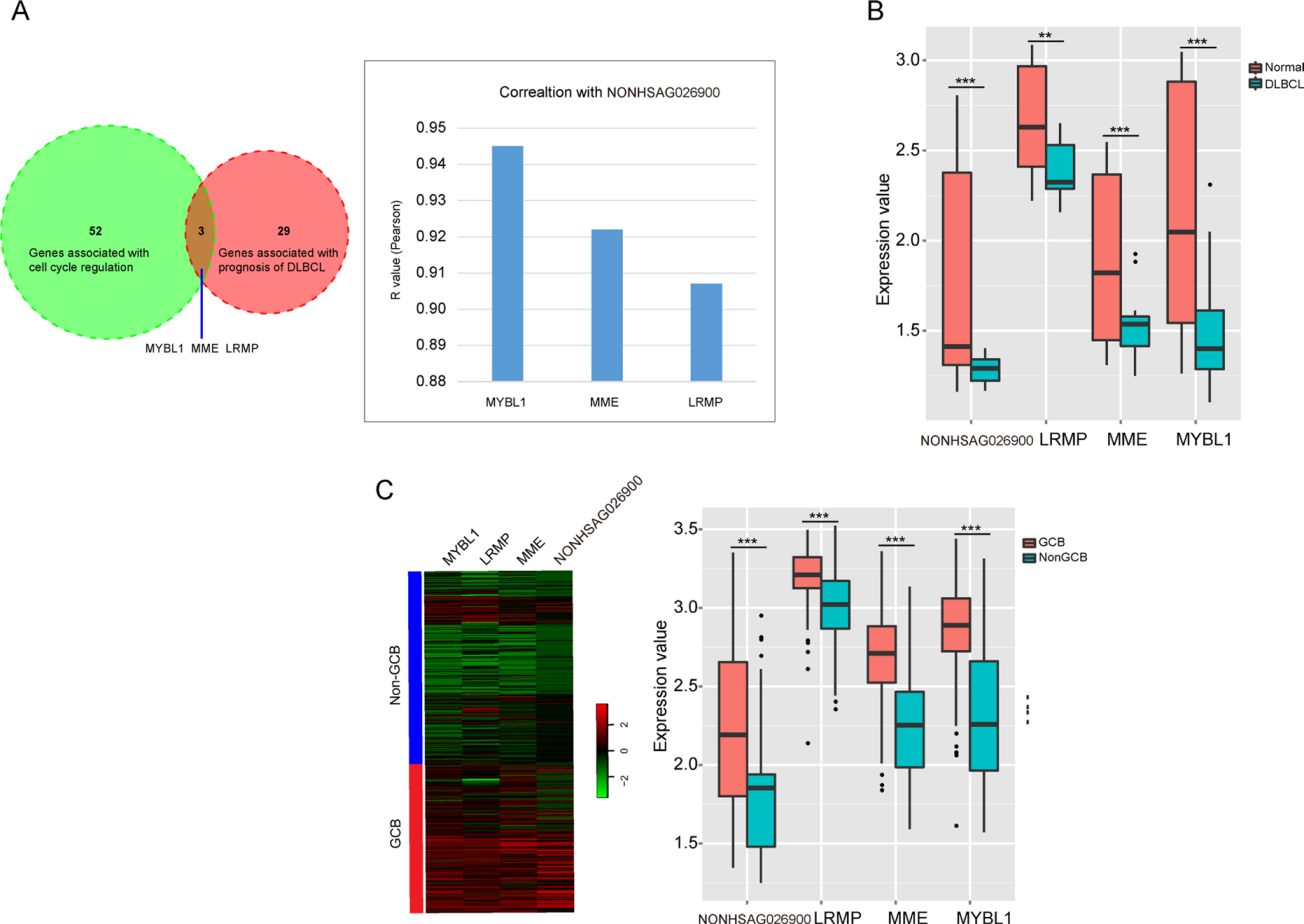
LncRNA NONHSAG026900 functional prediction and validation (**A**) A Venn diagram showed the overlap between the genes associated with cell cycle regulation and the genes correlated with prognosis of DLBCL, and identified the three overlapping genes; the bar plot showed the correlation between the three overlapping genes and NONHSAG026900 (Pearson *r* > 0.9, *P* < 0.001); (**B**) Box plots showed that the three protein coding-genes (MYBL1, MME and LRMP) were significantly down-regulated in DLBCL compared to normal tissues in patients from GSE12453 (*P* < 0.001, *t*-test method), in addition to NONHSAG026900; (**C**) Microarray gene expression heatmap of the four genes (MYBL1, MME, LRMP and NONHSAG026900) between GCB and non-GCB subgroups in GSE11318. The expression values of the four genes were significantly higher in the GCB than non-GCB subgroup (*P* < 0.001, *t*-test method) in the boxplot on the right.

To validate the predicted function of NONHSAG026900 in cell proliferation was involved in its prognostic power, we compared the 55 protein-coding genes around NONHSAG026900 correlated with cell cycle regulation and 32 genes reported to be associated with prognosis of DLBCL. We discovered three common protein-coding genes (MYBL1, MME and LRMP) in the two groups (Figure 6A). Next, we investigated whether there was any interaction between MYBL1, MME, LRMP and NONHSAG026900. The expression of NONHSAG026900 was positively correlated with the mRNA expression values of MYBL1, MME and LRMP in the DLBCL tissues from GSE12453 (Figure 6A, Pearson *r* > 0.9, *P* < 0.001). Moreover, the expressions of these three protein-coding genes were also significantly down-regulated in DLBCL samples when compared with normal samples (*P* < 0.05, Figure 6B), which agreed with the results from NONHSAG026900 outlined above. Meanwhile, we analyzed the expressions of MYBL1, MME and LRMP between the GCB and non-GCB subgroups in patients from GSE11318. The three genes were significantly down-regulated in the non-GCB compared to the GCB subgroup (*P* < 0.001, Figure 6C), which was also in line with the NONHSAG026900 expression distribution. Interestingly, some studies reported that these three genes could be prognostic biomarkers for DLBCL because they stimulate cell proliferation and differentiation [23, 25–30]. Taken together, our results suggested that the prognostic power of NONHSAG026900 was related to its predicted role in cell proliferation regulation like the three protein-coding biomarkers in patients with DLCBL.

## DISCUSSION

DLBCL is one of the most pathologically heterogeneous cancers, and a large number of patients relapse within 2–3 years after primary chemotherapy [31]. Therefore, it is significantly important to discover an effective prognostic marker to apply into clinical practice. Some research revealed that many lncRNAs were involved in the process of tumor, including cancer cell proliferation, adhesion, migration, and cell cycle [32–34]. And the correlation between lncRNAs and cancer significance has shown that some lncRNAs could be utilized as biomarkers for prognosis or diagnosis [35–37] . Meng Zhou etc. identified a 17-lncRNA signature to classify subtype and predict prognosis in 1,118 patients with DLBCL [38]; Yuling Yan etc. reported a lncRNA HOTAIR could predict a poor prognosis, which promotes cell proliferation and is correlated with tumor size and IPI [39]; Wei Peng etc. demonstrated that the lncRNA LUNAR1 and PEG10 as indicators of poor survival rate may play a key oncogene role promoting cell proliferation for DLBCL patients [40, 41]. Despite the progress has been made in the prognostic biomarkers of lncRNAs in DLBCL patients, further research needs to be done to discover more precise forecast genes.

In our study, we re-analyzed 7 microarray datasets to examine the pathogenesis of DLBCL. And we found the lncRNA NONHSAG026900 was significantly lower expression in DLBCL cells compared with the five differentiated stages of normal B cells (*P* < 0.05). We also discovered that the expression of NONHSAG026900 was significantly higher in GCB-DLBCL patients than non-GCB-DLBCL patients (*P* = 0.000). Because patients with GCB-DLBCL have a more favorable outcome than those with non-GCB-DLBCL [42], we inferred that NONHSAG026900 might be used as a biomarker to predict the prognosis of DLBCL patients. Subsequently, we confirmed our discovery in patients with CHOP or R-CHOP regimens from another four GEO datasets (GSE10846, GSE53786, GSE23501, and GSE31312). Kaplan-Meier analysis suggested that patients with low expression of NONHSAG026900 had significantly higher risk and shorter 5-year OS or PFS ratios than those from the high value group. In addition, we determined the correlation between NONHSAG026900 and IPI, and found that the NONHSAG026900 could add to the predictive power of IPI as an independent biomarker from multivariate Cox regression analysis. To the best of our knowledge, this is the first report that showed that NONHSAG026900 was a favorable predictor of survival in a large group of DLBCL patients.

The cell cycle is a critical factor that controls cell division during cancer development. MYBL1, MME and LRMP, as key regulators of the cell cycle, have been identified as potential therapeutic targets for cancer. MYBL1, which is located in chromosome region 8q22, could be involved in recurrent translocations in malignant lymphoma [28]. MYBL1 is highly expressed in Burkitt's lymphoma cells, some chronic lymphocytic leukemia, and a small subset of human neoplastic B-cells and stimulates the proliferation and differentiation as a member of the Myb oncogene family of transcription factors [29, 43, 44]. It is specifically induced in proliferating centroblasts and acts as a specific marker for the proliferation of centroblasts [30]. However, Golay etc. discovered that MYBL1 mRNA was very weak or negative in DLBCL B-cell lines compared with normal tonsillar buoyant B cells [44], which demonstrated that the similar change trend with our analysis in Figure 6B. MME, is a proliferation blocker, can cleave signal peptides at the cell surface to affect cell proliferation and differentiation, and acts as an acute lymphocytic leukemia antigen [26, 27]. In our research, the expression of MME was significantly down-regulated in the non-GCB compared to the GCB subgroup. Therefore, improved expression of MME was associated with a favorable outcome in DLBCL patients. The lymphoid-restricted membrane protein (LRMP), is an endoplasmic reticulum-associated protein. We found higher mRNA levels of LRMP in the GCB- compared to the non-GCB-subtype of DLBCL, which demonstrated that it predicted and supported the aggressive behavior of the non-GCB subtype of DLBCL [23, 25]. In this study, we discovered that NONHSAG026900 was clustered with these three protein-coding genes into one functional module for cell cycle regulation by gene co-expression network analysis. NONHSAG026900 was significantly down regulated in DLBCL samples, especially in the non-GCB subtype, which suggested that its presence could inhibit cell cycle activity to restrain tumor growth and thereby predict the prognosis of patients with DLBCL.

We agreed that the biological validation is in some way weak in this study. We understand that it is better to reveal the potential lncRNA transcriptional mechanism by the examination of the methylated sites in corresponding cancer cells and the expression of lncRNA NONHSAG026900. However, in the present study, we mainly focused on exploring an analysis method with bioinformatics tools to reveal the mechanism of lncRNA NONHSAG026900 with lower expression in DLBCL patients. And the further efforts in the next study will be paid to achieve the corresponding experiment above and validate the discoveries about the expression and function of this lncRNA with modern empirical method of molecular biology.

Generally, our results demonstrated that decreased NONHSAG026900 expression was observed frequently in DLBCL and could be identified as a novel biomarker for diagnosis and an independent factor for predicting prognosis of patients with DLBCL. This suggested that NONHSAG026900 might enhance tumor suppression as an indicator of favorable survival ratio, and function as a positive prognostic factor for patients with DLBCL. Moreover, a deeper understanding of the mechanisms of NONHSAG026900 in DLBCL will promote the development of NONHSAG026900-directed diagnostic, prognostic and therapeutic agents against this malignancy.

## MATERIALS AND METHODS

### Microarray data and patient information

We obtained published Affymetrix platform HG-U133A Plus 2.0 microarray data sets from the Gene Expression Omnibus (GEO) database (accession numbers: GSE12453, GSE56315, GSE11318, GSE23501, GSE53786, GSE10846, and GSE31312). The microarray data assessed the gene expression profiles of 36 samples consisting of 5 naïve B-cell samples, 5 centroblast samples, 5 centrocyte samples, 5 memory B cell samples, 5 plasma cell samples, and 11 DLBCL samples in GSE12453. Similarly, there were 6 naïve B-cell samples, 7 centroblast samples, 7 centrocyte samples, 6 memory B cell samples, 7 plasma cell samples, and 74 DLBCL samples in the validated cohort GSE56315. In the other five independent cohorts, a total of 170, 420, 117, 69, and 484 patients were from GSE11318, GSE10846, GSE53786, GSE23501, and GSE31312, respectively. The number of patients with CHOP, R-CHOP like, or other regimens were 164, 0 and 6 in GSE11318. GSE10846 was composed of 181 patients with CHOP-like regimen, 233 patients with R-CHOP regimen, and 6 patients with other regimens, while the number of patients with CHOP, R-CHOP like or other regimens was 45, 71 and 1 in GSE53786, and 1, 64, and 4 in GSE23501, respectively. However, 484 patients with R-CHOP like regimen only were selected from the GSE31312 cohort.

### Repurposing microarray data

We quantified the expression levels of messenger RNAs and long non-coding RNAs by the re-annotation of Affymetrix microarray probes using the Non-coding RNA Function Annotation Server (ncFANs) [45]. We submitted 7 microarray datasets with a CEL format to ncFANs and acquired lncRNA and protein-coding gene expression values (log2-transformed) using R. Meanwhile, we used the Limma statistical package to implement the Robust Multichip Average (RMA) analysis, Student's *t*-test, one-way ANOVA (paired with an F test) and Benjamini Hochberg (BH) false discovery rate (FDR) correction. Therefore, we considered genes with a Fold-Change > 2 and BH FDR-adjusted *p*-values < 0.05 as those with different expressions.

### Functional enrichment of protein-coding genes

We utilized the DAVID Bioinformatics Tool [46] to perform the functional enrichment of target protein-coding genes. We identified the biological processes related to protein-coding genes by conducting gene ontology analysis with this tool. In our study, we defined *p*-values < 0.05 as a significance threshold. Finally, the Enrichment Map plugin [47] for Cytoscape [48] was used to visualize the biological process organization.

### Functional analysis of lncRNAs

We utilized gene expression data of 36 tissue samples from GSE12453 to produce a co-expression network including non-coding and protein-coding genes. Then we chose both protein-coding and non-coding genes with different expressions to construct a co-expression network. We evaluated the relationship of each gene pair (coding-coding, coding-lncRNA, and lncRNA-lncRNA gene pairs) with a Pearson's correlation coefficient or a Spearman Rank correlation coefficient. We utilized Fisher's asymptotic test to calculate the *p*-values of the correction coefficients for each gene pair, and then adjusted this with a Bonferroni multiple testing correction. Next, we developed the coding-lncRNA co-expression network by selecting co-expression gene pairs with *p*-values < 0.01. Based on the coding-lncRNA co-expression network, we predicted the functions of lncRNAs by module-based and hub-based methods embedded in ncFANs. We identified modules of co-expressed genes in the coding-lncRNA co-expression network by the Markov cluster algorithm (MCL) in the module based method. We further analyzed the protein-coding genes in the same module as the lncRNAs by gene ontology analysis in order to predict the functions of lncRNAs in this module. Subsequently, the co-expression network was parsed into several subnetworks with the hub-based method, which consisted of some protein coding genes around a central lncRNA. We inferred the function of the corresponding lncRNA by the functional enrichment of these connected protein-coding genes. In our analysis, we only reserved the function enrichments with *p*-values < 0.01.

### Statistical analysis

Pearson Chi-square tests and the student's *t* test analysis of variance were utilized to analyze statistical differences in demographic and clinical characteristics. The paired student-test compared distributive differences of NONHSAG026900 expression between germinal center B-cell like (GCB) and non-GCB subgroups. Receiver-operating characteristic (ROC) curve analysis was used to visualize the specificity and sensitivity of the biomarker NONHSAG026900 in predicting prognosis of patients with DLBCL. The performance was quantified by the area under the ROC curve. The correlation between NONHSAG026900 expression and overall survival (OS) or progression-free survival (PFS) of patients was assessed by univariate Cox regression analysis. Survival differences between low and high value groups in each set were evaluated by the Kaplan-Meier estimate, and compared by the log-rank test. To explore whether the predictive power of NONHSAG026900 was independent of IPI scores, multivariate Cox regression analysis and data stratification analysis were conducted in our study. Statistical analysis was performed with SPSS 13.0, and presented with GraphPad Prism 5.0 and R3.2.2 software. Results were considered statistically significant at *P* < 0.05.

## SUPPLEMENTARY MATERIALS FIGURES AND TABLES








