# Supplementary material for: The long non-coding RNA NONHSAG026900 predicts prognosis as a favorable biomarker in patients with diffuse large B-cell lymphoma
Source: Oncotarget. 2017 Mar 13;8(21):34374–86. doi: 10.18632/oncotarget.16163 (PMC5470975; doi:10.18632/oncotarget.16163)
Supplement: Supplementary file 1 [file oncotarget-08-34374-s001.pdf]

The long non-coding RNA NONHSAG026900 predicts prognosis as a favorable biomarker in patients with diffuse large B-cell lymphoma

Supplementary Materials

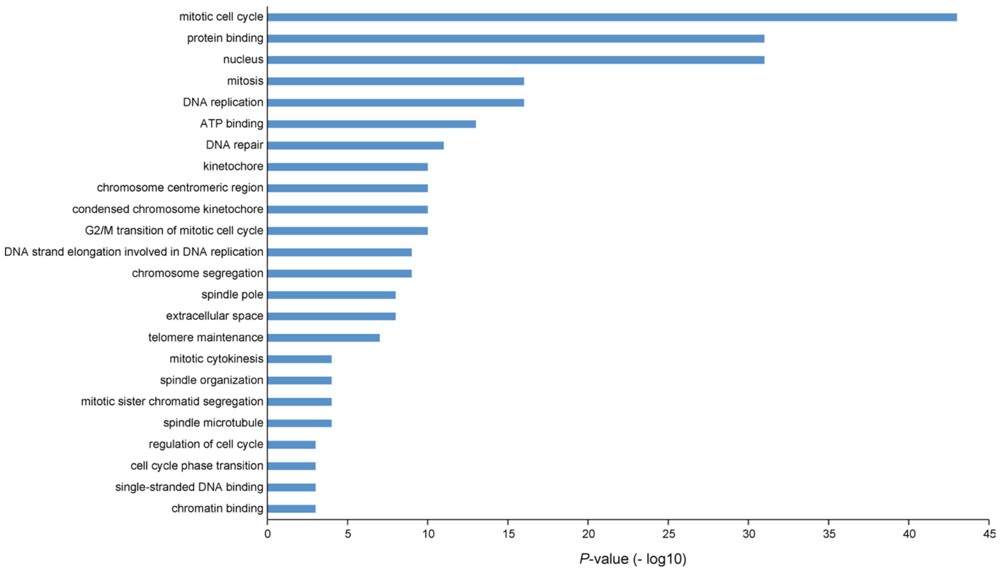

Supplementary Figure 1: NONHSAG026900 functions were predicted with a module-based analysis. Functional enrichment results of the module including NONHSAG026900.

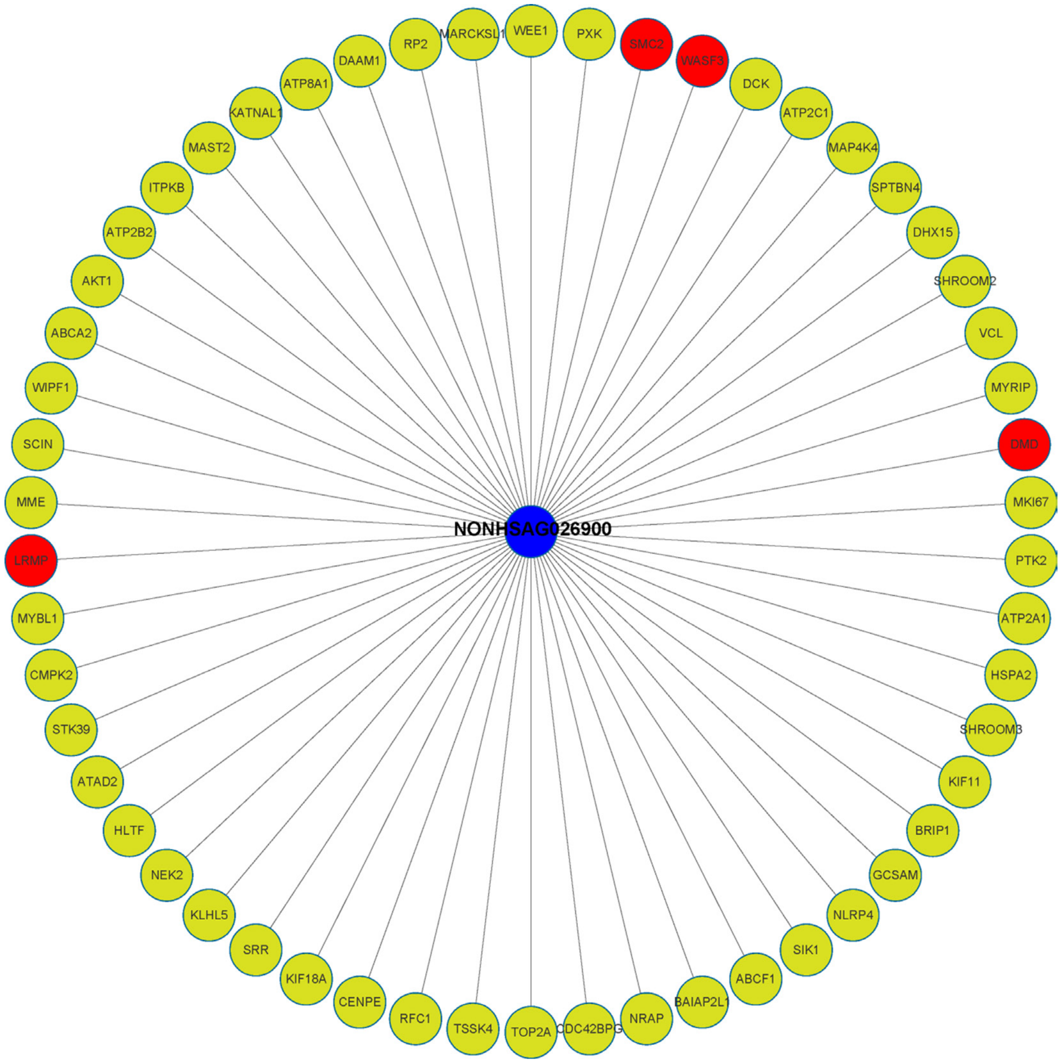

Supplementary Figure 2: NONHSAG026900 functions were predicted with a hub-based analysis. The NONHSAG026900-centered subnetwork. Genes around NONHSAG02900 were protein-coding genes, while red genes were protein complex-coding genes.

Supplementary Table 1: 226 protein-coding genes with significantly different expression between normal and DLBCL samples. See Supplementary_Table_1

Supplementary Table 2: Functional enrichment results of the module for NONHSAG026900. See Supplementary_Table_2

Supplementary Table 3: Functional enrichment results of the hub for NONHSAG026900. See Supplementary_Table_3
